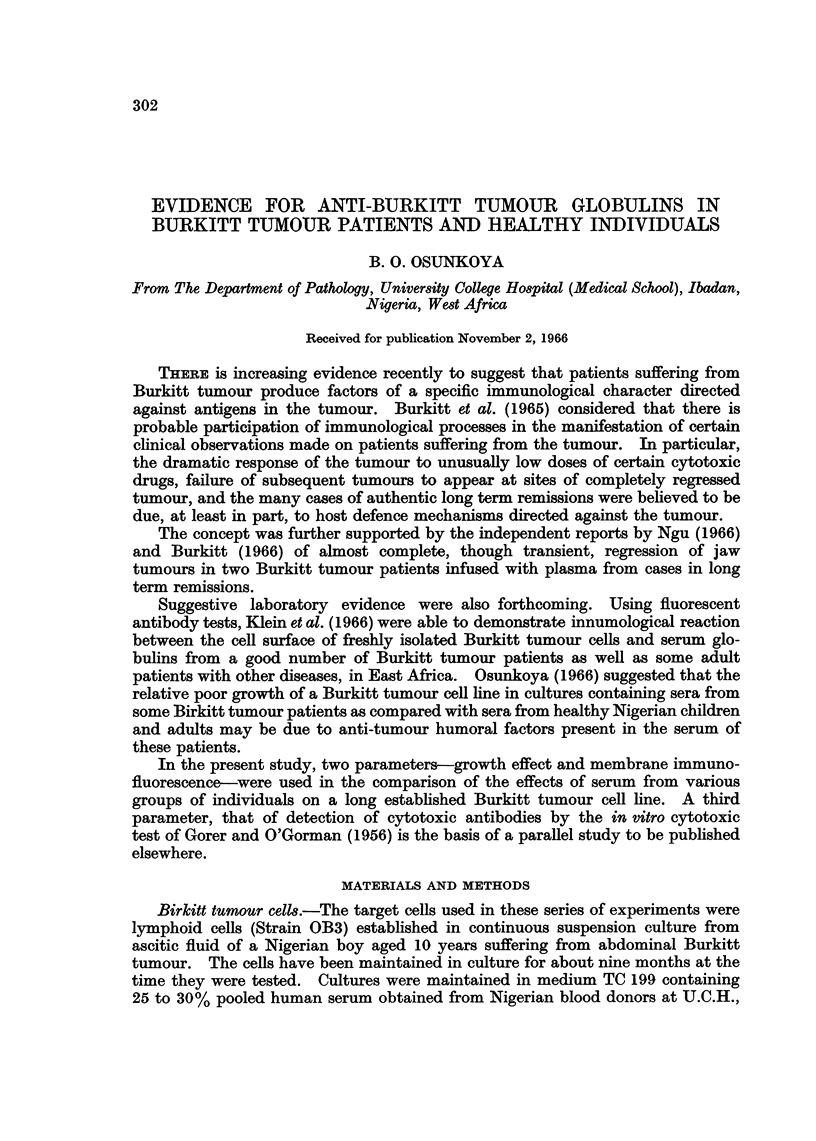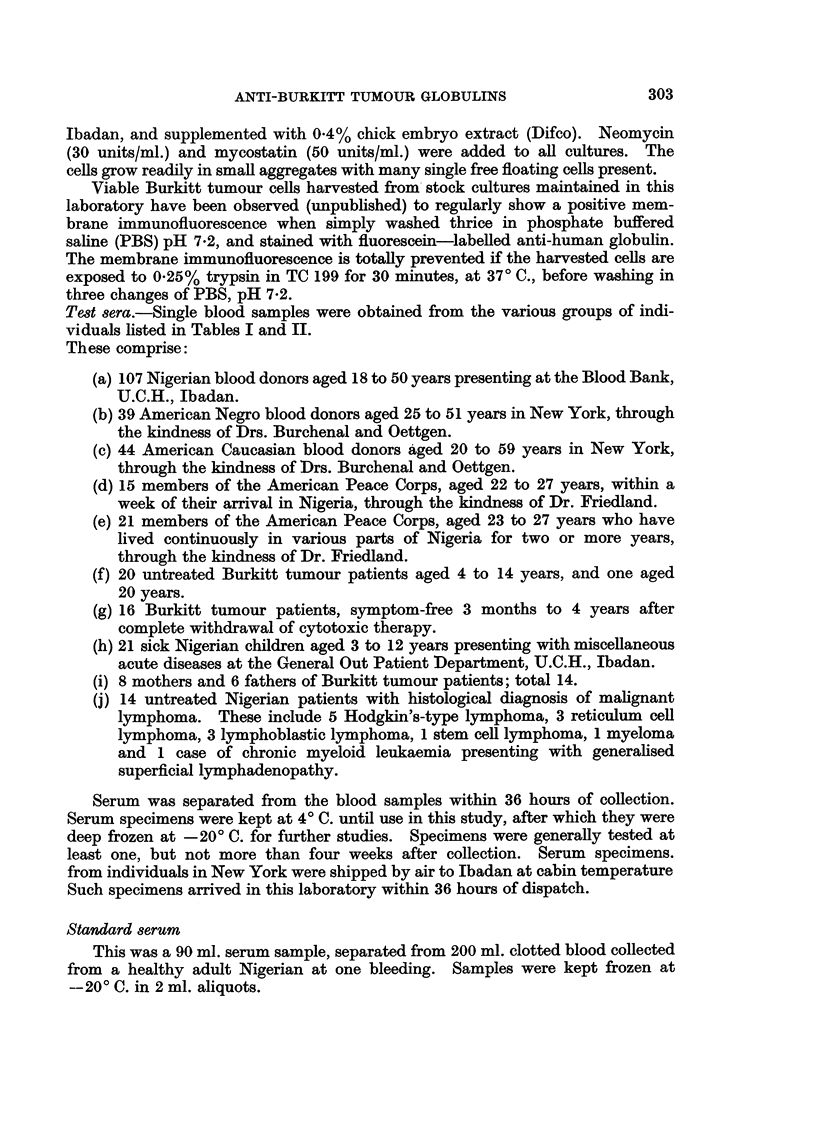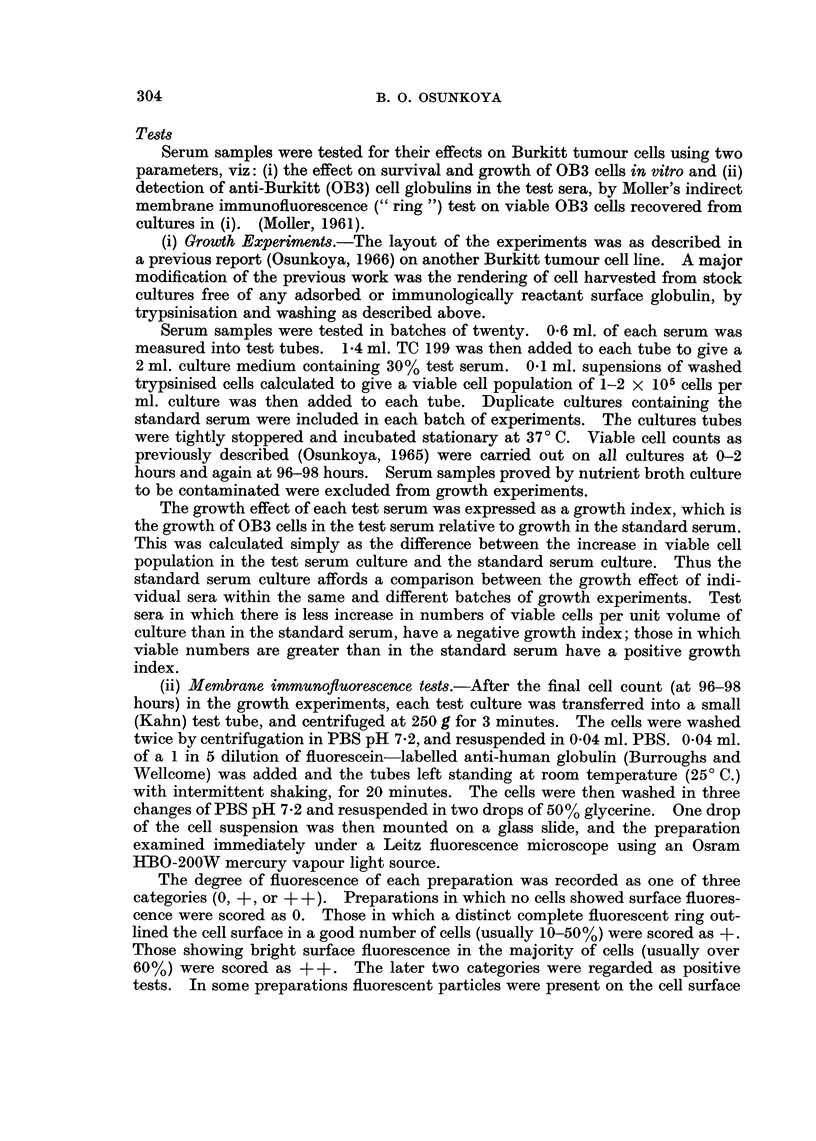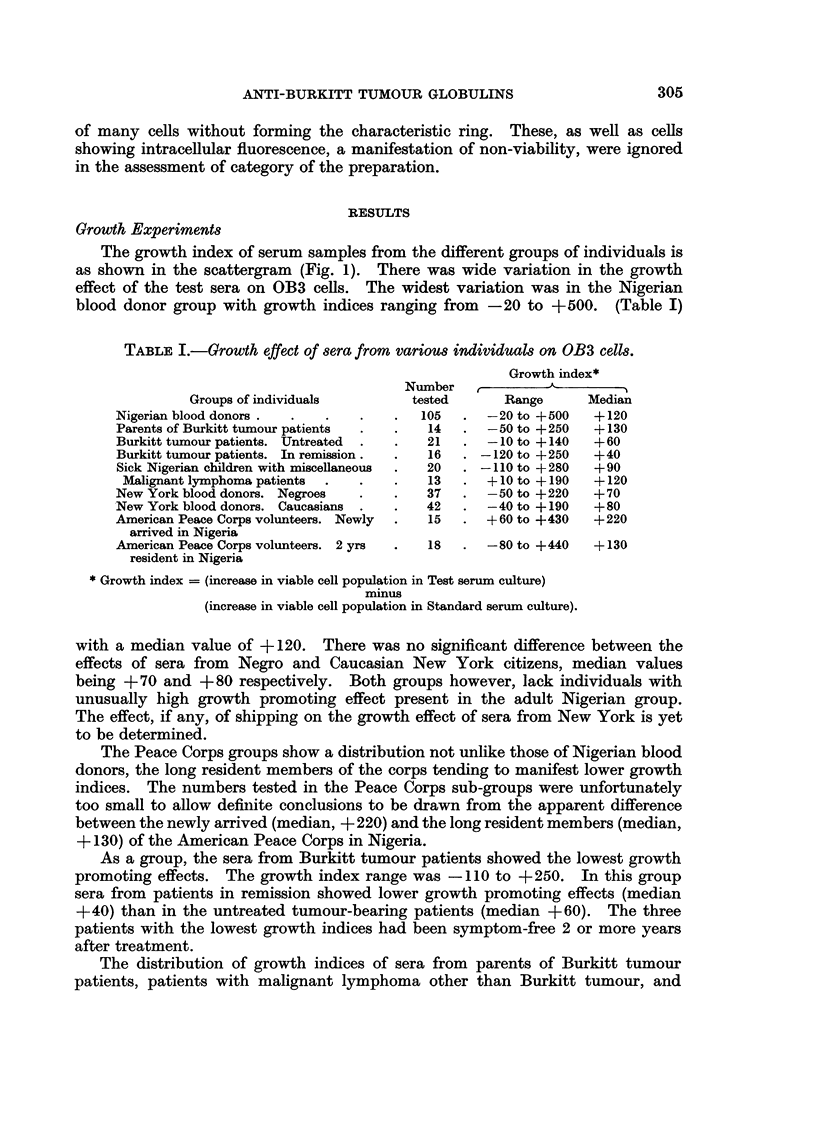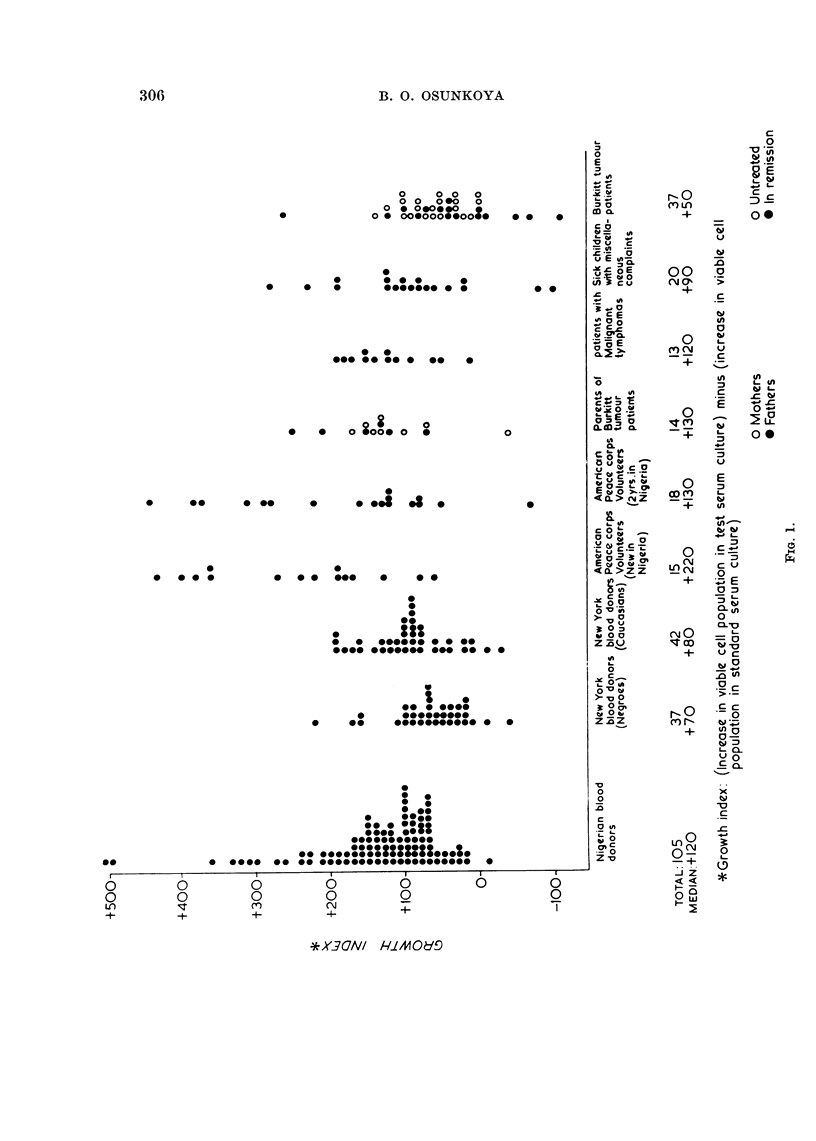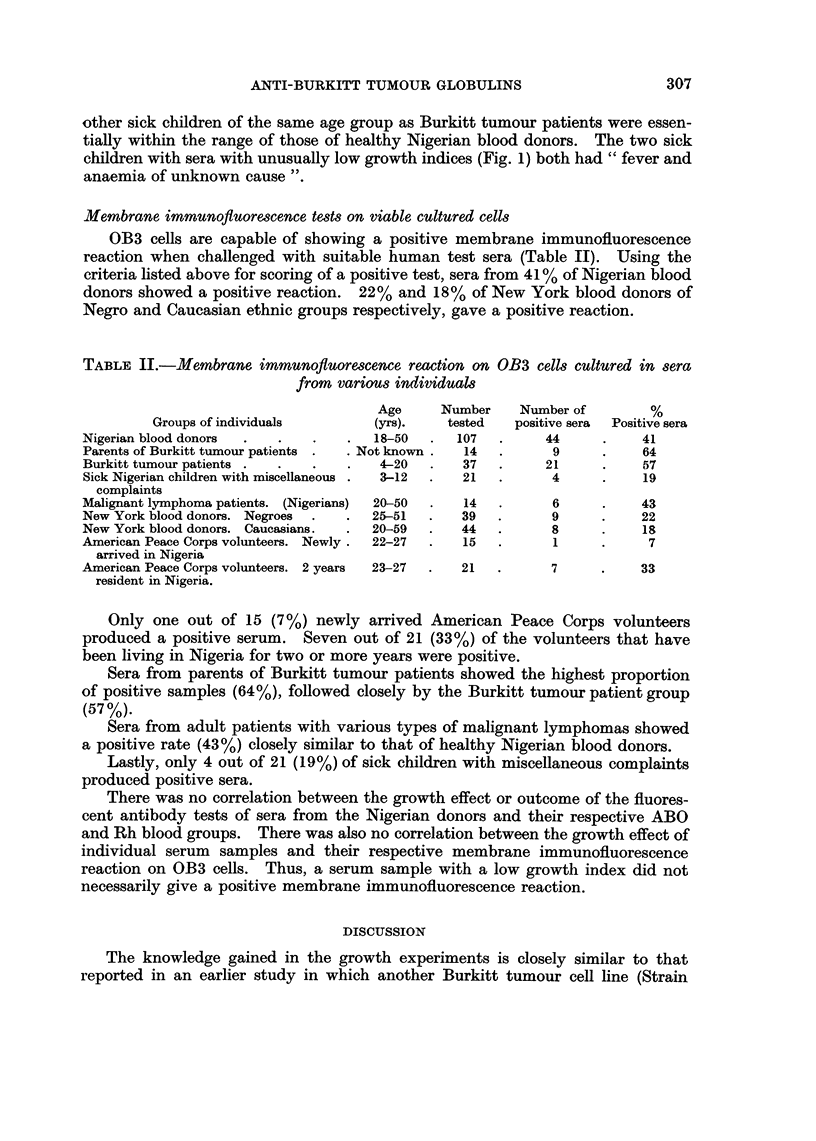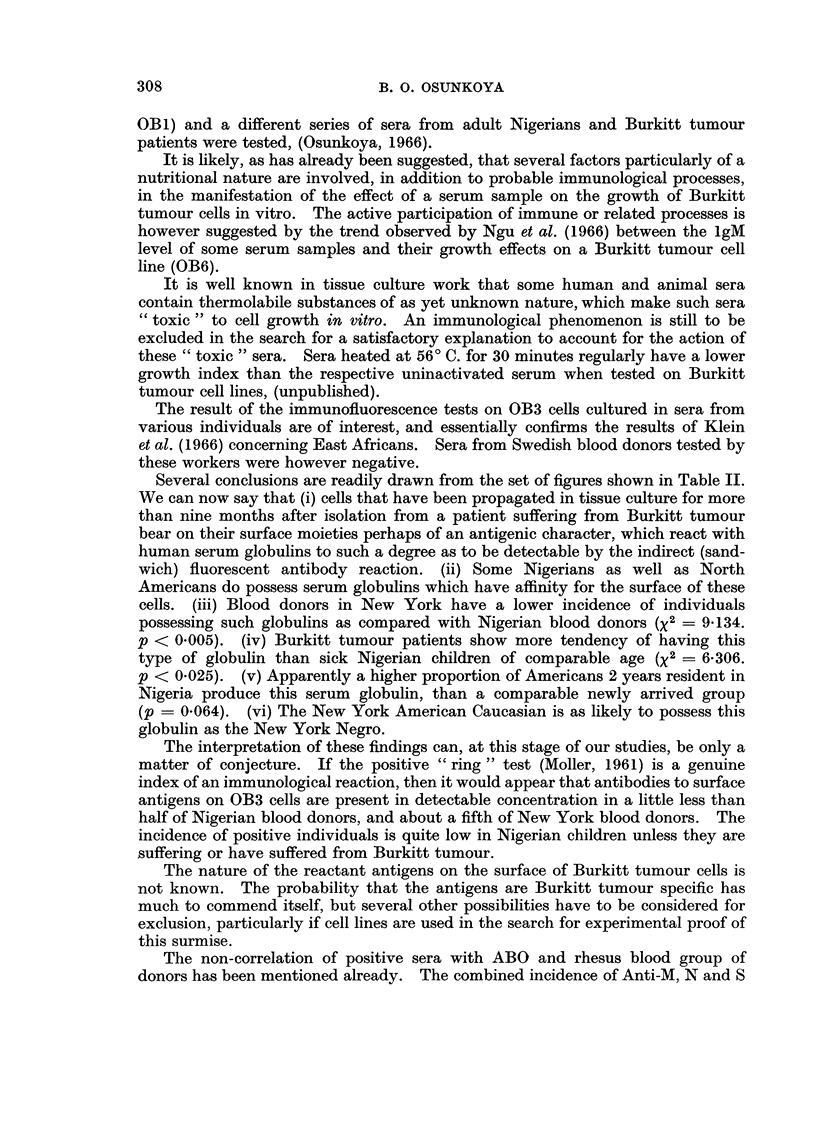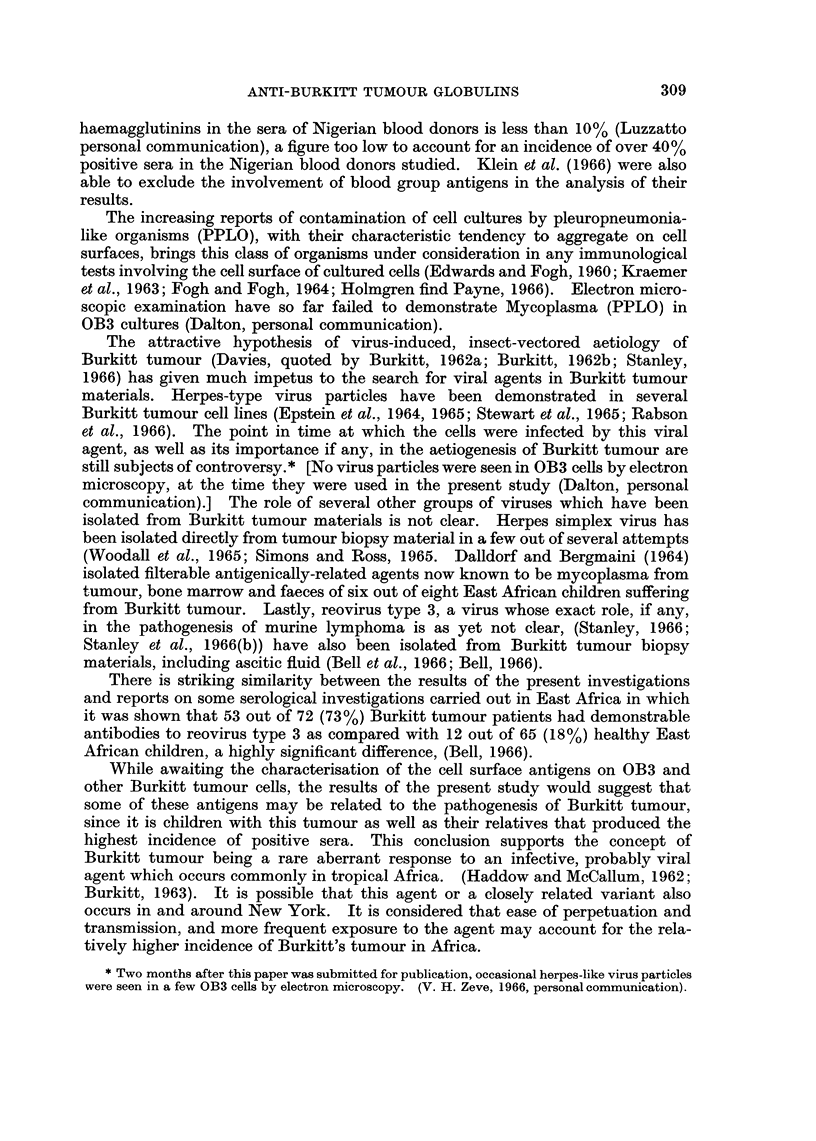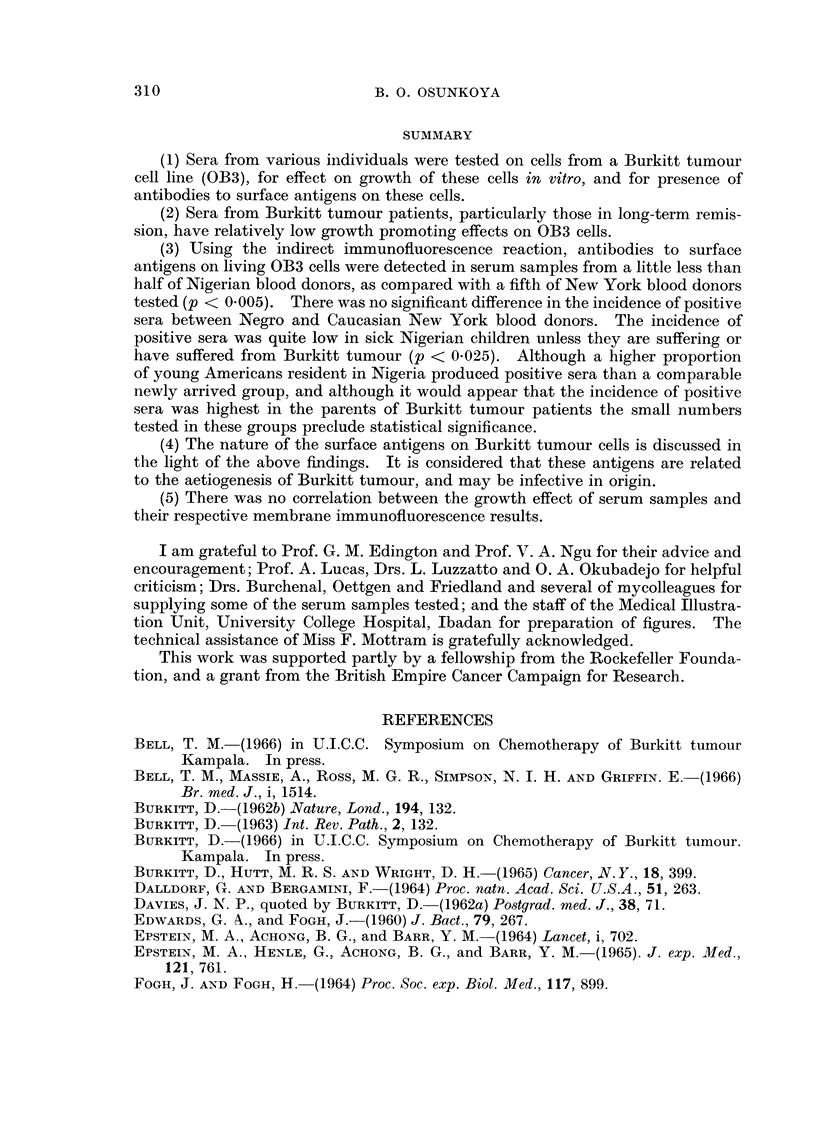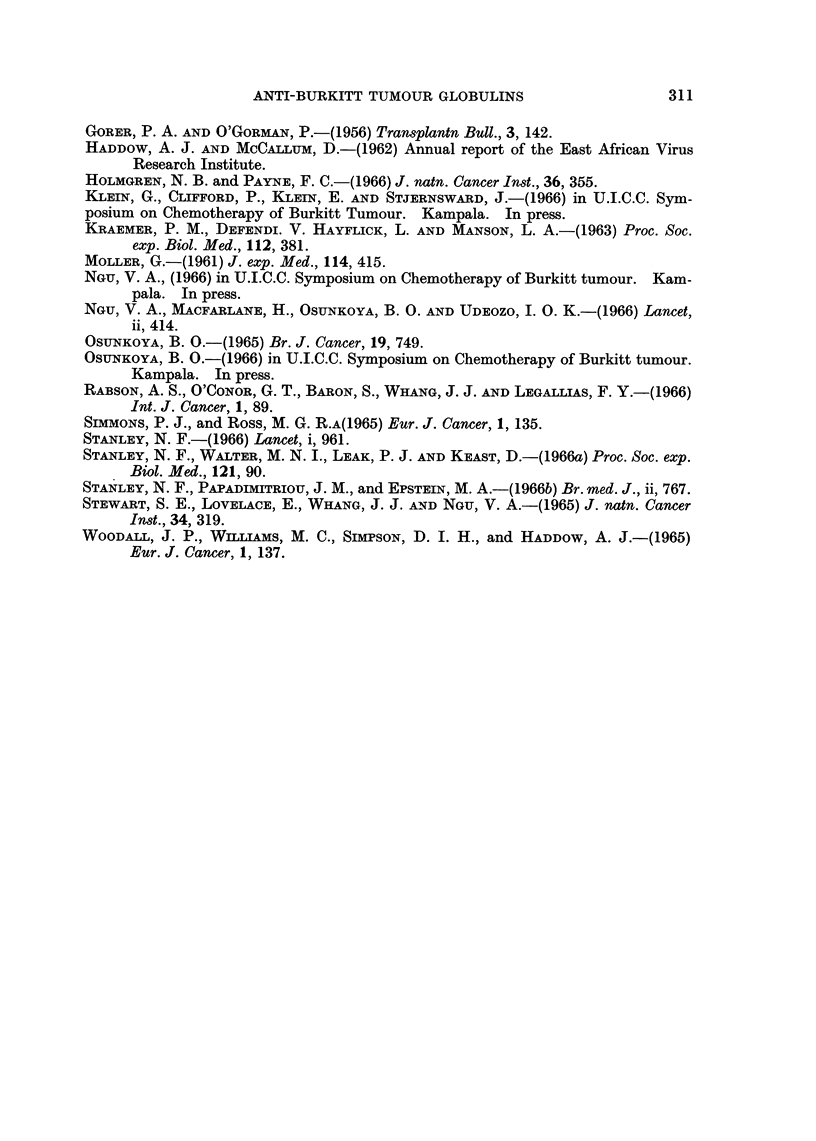# Evidence for anti-Burkitt tumour globulins in Burkitt tumour patients and healthy individuals.

**DOI:** 10.1038/bjc.1967.33

**Published:** 1967-06

**Authors:** B. O. Osunkoya


### EVIDENCE FOR ANTI-BURKITT TUMOUR GLOBULINS IN BURKITT TUMOUR PATIENTS AND HEALTHY INDIVIDUALS

B. O. OSUNKOYA

*From The Department of Pathology, University College Hospital (Medical School), Ibadan, Nigeria, West Africa*

Received for publication November 2, 1966

THERE is increasing evidence recently to suggest that patients suffering from Burkitt tumour produce factors of a specific immunological character directed against antigens in the tumour. Burkitt *et al.* (1965) considered that there is probable participation of immunological processes in the manifestation of certain clinical observations made on patients suffering from the tumour. In particular, the dramatic response of the tumour to unusually low doses of certain cytotoxic drugs, failure of subsequent tumours to appear at sites of completely regressed tumour, and the many cases of authentic long term remissions were believed to be due, at least in part, to host defence mechanisms directed against the tumour.

The concept was further supported by the independent reports by Ngu (1966) and Burkitt (1966) of almost complete, though transient, regression of jaw tumours in two Burkitt tumour patients infused with plasma from cases in long term remissions.

Suggestive laboratory evidence were also forthcoming. Using fluorescent antibody tests, Klein *et al.* (1966) were able to demonstrate innumological reaction between the cell surface of freshly isolated Burkitt tumour cells and serum globulins from a good number of Burkitt tumour patients as well as some adult patients with other diseases, in East Africa. Osunkoya (1966) suggested that the relative poor growth of a Burkitt tumour cell line in cultures containing sera from some Birkitt tumour patients as compared with sera from healthy Nigerian children and adults may be due to anti-tumour humoral factors present in the serum of these patients.

In the present study, two parameters—growth effect and membrane immunofluorescence—were used in the comparison of the effects of serum from various groups of individuals on a long established Burkitt tumour cell line. A third parameter, that of detection of cytotoxic antibodies by the *in vitro* cytotoxic test of Gorer and O'Gorman (1956) is the basis of a parallel study to be published elsewhere.

### MATERIALS AND METHODS

*Birkitt tumour cells.*—The target cells used in these series of experiments were lymphoid cells (Strain OB3) established in continuous suspension culture from ascitic fluid of a Nigerian boy aged 10 years suffering from abdominal Burkitt tumour. The cells have been maintained in culture for about nine months at the time they were tested. Cultures were maintained in medium TC 199 containing 25 to 30% pooled human serum obtained from Nigerian blood donors at U.C.H.,

Ibadan, and supplemented with 0·4% chick embryo extract (Difco). Neomycin (30 units/ml.) and mycostatin (50 units/ml.) were added to all cultures. The cells grow readily in small aggregates with many single free floating cells present.

Viable Burkitt tumour cells harvested from stock cultures maintained in this laboratory have been observed (unpublished) to regularly show a positive membrane immunofluorescence when simply washed thrice in phosphate buffered saline (PBS) pH 7·2, and stained with fluorescein—labelled anti-human globulin. The membrane immunofluorescence is totally prevented if the harvested cells are exposed to 0·25% trypsin in TC 199 for 30 minutes, at 37° C., before washing in three changes of PBS, pH 7·2.

*Test sera*.—Single blood samples were obtained from the various groups of individuals listed in Tables I and II.

These comprise:

(a) 107 Nigerian blood donors aged 18 to 50 years presenting at the Blood Bank, U.C.H., Ibadan.
(b) 39 American Negro blood donors aged 25 to 51 years in New York, through the kindness of Drs. Burchenal and Oettgen.
(c) 44 American Caucasian blood donors aged 20 to 59 years in New York, through the kindness of Drs. Burchenal and Oettgen.
(d) 15 members of the American Peace Corps, aged 22 to 27 years, within a week of their arrival in Nigeria, through the kindness of Dr. Friedland.
(e) 21 members of the American Peace Corps, aged 23 to 27 years who have lived continuously in various parts of Nigeria for two or more years, through the kindness of Dr. Friedland.
(f) 20 untreated Burkitt tumour patients aged 4 to 14 years, and one aged 20 years.
(g) 16 Burkitt tumour patients, symptom-free 3 months to 4 years after complete withdrawal of cytotoxic therapy.
(h) 21 sick Nigerian children aged 3 to 12 years presenting with miscellaneous acute diseases at the General Out Patient Department, U.C.H., Ibadan.
(i) 8 mothers and 6 fathers of Burkitt tumour patients; total 14.
(j) 14 untreated Nigerian patients with histological diagnosis of malignant lymphoma. These include 5 Hodgkin's-type lymphoma, 3 reticulum cell lymphoma, 3 lymphoblastic lymphoma, 1 stem cell lymphoma, 1 myeloma and 1 case of chronic myeloid leukaemia presenting with generalised superficial lymphadenopathy.

Serum was separated from the blood samples within 36 hours of collection. Serum specimens were kept at 4° C. until use in this study, after which they were deep frozen at −20° C. for further studies. Specimens were generally tested at least one, but not more than four weeks after collection. Serum specimens. from individuals in New York were shipped by air to Ibadan at cabin temperature Such specimens arrived in this laboratory within 36 hours of dispatch.

*Standard serum*

This was a 90 ml. serum sample, separated from 200 ml. clotted blood collected from a healthy adult Nigerian at one bleeding. Samples were kept frozen at −20° C. in 2 ml. aliquots.

*Tests*

Serum samples were tested for their effects on Burkitt tumour cells using two parameters, viz: (i) the effect on survival and growth of OB3 cells *in vitro* and (ii) detection of anti-Burkitt (OB3) cell globulins in the test sera, by Moller's indirect membrane immunofluorescence (" ring ") test on viable OB3 cells recovered from cultures in (i). (Moller, 1961).

(i) *Growth Experiments.*—The layout of the experiments was as described in a previous report (Osunkoya, 1966) on another Burkitt tumour cell line. A major modification of the previous work was the rendering of cell harvested from stock cultures free of any adsorbed or immunologically reactant surface globulin, by trypsinisation and washing as described above.

Serum samples were tested in batches of twenty. 0·6 ml. of each serum was measured into test tubes. 1·4 ml. TC 199 was then added to each tube to give a 2 ml. culture medium containing 30% test serum. 0·1 ml. supensions of washed trypsinised cells calculated to give a viable cell population of $1–2 \times 10^5$ cells per ml. culture was then added to each tube. Duplicate cultures containing the standard serum were included in each batch of experiments. The cultures tubes were tightly stoppered and incubated stationary at 37° C. Viable cell counts as previously described (Osunkoya, 1965) were carried out on all cultures at 0–2 hours and again at 96–98 hours. Serum samples proved by nutrient broth culture to be contaminated were excluded from growth experiments.

The growth effect of each test serum was expressed as a growth index, which is the growth of OB3 cells in the test serum relative to growth in the standard serum. This was calculated simply as the difference between the increase in viable cell population in the test serum culture and the standard serum culture. Thus the standard serum culture affords a comparison between the growth effect of individual sera within the same and different batches of growth experiments. Test sera in which there is less increase in numbers of viable cells per unit volume of culture than in the standard serum, have a negative growth index; those in which viable numbers are greater than in the standard serum have a positive growth index.

(ii) *Membrane immunofluorescence tests.*—After the final cell count (at 96–98 hours) in the growth experiments, each test culture was transferred into a small (Kahn) test tube, and centrifuged at 250 *g* for 3 minutes. The cells were washed twice by centrifugation in PBS pH 7·2, and resuspended in 0·04 ml. PBS. 0·04 ml. of a 1 in 5 dilution of fluorescein—labelled anti-human globulin (Burroughs and Wellcome) was added and the tubes left standing at room temperature (25° C.) with intermittent shaking, for 20 minutes. The cells were then washed in three changes of PBS pH 7·2 and resuspended in two drops of 50% glycerine. One drop of the cell suspension was then mounted on a glass slide, and the preparation examined immediately under a Leitz fluorescence microscope using an Osram HBO-200W mercury vapour light source.

The degree of fluorescence of each preparation was recorded as one of three categories (0, +, or ++). Preparations in which no cells showed surface fluorescence were scored as 0. Those in which a distinct complete fluorescent ring outlined the cell surface in a good number of cells (usually 10–50%) were scored as +. Those showing bright surface fluorescence in the majority of cells (usually over 60%) were scored as ++. The later two categories were regarded as positive tests. In some preparations fluorescent particles were present on the cell surface of many cells without forming the characteristic ring. These, as well as cells showing intracellular fluorescence, a manifestation of non-viability, were ignored in the assessment of category of the preparation.

### RESULTS

### *Growth Experiments*

The growth index of serum samples from the different groups of individuals is as shown in the scattergram (Fig. 1). There was wide variation in the growth effect of the test sera on OB3 cells. The widest variation was in the Nigerian blood donor group with growth indices ranging from —20 to +500. (Table I)

TABLE I.—*Growth effect of sera from various individuals on OB3 cells.*

| Groups of individuals | Number tested | Growth index* Range | Median |
|---|---|---|---|
| Nigerian blood donors | 105 | −20 to +500 | +120 |
| Parents of Burkitt tumour patients | 14 | −50 to +250 | +130 |
| Burkitt tumour patients. Untreated | 21 | −10 to +140 | +60 |
| Burkitt tumour patients. In remission | 16 | −120 to +250 | +40 |
| Sick Nigerian children with miscellaneous | 20 | −110 to +280 | +90 |
| Malignant lymphoma patients | 13 | +10 to +190 | +120 |
| New York blood donors. Negroes | 37 | −50 to +220 | +70 |
| New York blood donors. Caucasians | 42 | −40 to +190 | +80 |
| American Peace Corps volunteers. Newly arrived in Nigeria | 15 | +60 to +430 | +220 |
| American Peace Corps volunteers. 2 yrs resident in Nigeria | 18 | −80 to +440 | +130 |

* Growth index = (increase in viable cell population in Test serum culture) minus (increase in viable cell population in Standard serum culture).

with a median value of +120. There was no significant difference between the effects of sera from Negro and Caucasian New York citizens, median values being +70 and +80 respectively. Both groups however, lack individuals with unusually high growth promoting effect present in the adult Nigerian group. The effect, if any, of shipping on the growth effect of sera from New York is yet to be determined.

The Peace Corps groups show a distribution not unlike those of Nigerian blood donors, the long resident members of the corps tending to manifest lower growth indices. The numbers tested in the Peace Corps sub-groups were unfortunately too small to allow definite conclusions to be drawn from the apparent difference between the newly arrived (median, +220) and the long resident members (median, +130) of the American Peace Corps in Nigeria.

As a group, the sera from Burkitt tumour patients showed the lowest growth promoting effects. The growth index range was —110 to +250. In this group sera from patients in remission showed lower growth promoting effects (median +40) than in the untreated tumour-bearing patients (median +60). The three patients with the lowest growth indices had been symptom-free 2 or more years after treatment.

The distribution of growth indices of sera from parents of Burkitt tumour patients, patients with malignant lymphoma other than Burkitt tumour, and

GROWTH INDEX*

+500

+400

+300

+200

+100

0

-100

Nigerian blood donors

New York blood donors (Negroes)

New York blood donors (Caucasians)

American Peace corps Volunteers (New in Nigeria)

American Peace corps Volunteers (2yrs. in Nigeria)

Parents of Burkitt tumour patients patients with Malignant lymphomas

Sick children with miscellaneous complaints

Burkitt tumour patients

TOTAL: 105 37 42 15 18 14 13 20 37

MEDIAN: +120 +70 +80 +220 +130 +130 +120 +90 +50

*Growth index: (Increase in viable cell population in test serum culture) minus (increase in viable cell population in standard serum culture)

o Mothers

● Fathers o Untreated

● In remission

FIG. 1.

other sick children of the same age group as Burkitt tumour patients were essentially within the range of those of healthy Nigerian blood donors. The two sick children with sera with unusually low growth indices (Fig. 1) both had " fever and anaemia of unknown cause ".

*Membrane immunofluorescence tests on viable cultured cells*

OB3 cells are capable of showing a positive membrane immunofluorescence reaction when challenged with suitable human test sera (Table II). Using the criteria listed above for scoring of a positive test, sera from 41% of Nigerian blood donors showed a positive reaction. 22% and 18% of New York blood donors of Negro and Caucasian ethnic groups respectively, gave a positive reaction.

TABLE II.—*Membrane immunofluorescence reaction on OB3 cells cultured in sera from various individuals*

| Groups of individuals | Age (yrs). | Number tested | Number of positive sera | % Positive sera |
|---|---|---|---|---|
| Nigerian blood donors | 18–50 | 107 | 44 | 41 |
| Parents of Burkitt tumour patients | Not known | 14 | 9 | 64 |
| Burkitt tumour patients | 4–20 | 37 | 21 | 57 |
| Sick Nigerian children with miscellaneous complaints | 3–12 | 21 | 4 | 19 |
| Malignant lymphoma patients. (Nigerians) | 20–50 | 14 | 6 | 43 |
| New York blood donors. Negroes | 25–51 | 39 | 9 | 22 |
| New York blood donors. Caucasians. | 20–59 | 44 | 8 | 18 |
| American Peace Corps volunteers. Newly arrived in Nigeria | 22–27 | 15 | 1 | 7 |
| American Peace Corps volunteers. 2 years resident in Nigeria. | 23–27 | 21 | 7 | 33 |

Only one out of 15 (7%) newly arrived American Peace Corps volunteers produced a positive serum. Seven out of 21 (33%) of the volunteers that have been living in Nigeria for two or more years were positive.

Sera from parents of Burkitt tumour patients showed the highest proportion of positive samples (64%), followed closely by the Burkitt tumour patient group (57%).

Sera from adult patients with various types of malignant lymphomas showed a positive rate (43%) closely similar to that of healthy Nigerian blood donors.

Lastly, only 4 out of 21 (19%) of sick children with miscellaneous complaints produced positive sera.

There was no correlation between the growth effect or outcome of the fluorescent antibody tests of sera from the Nigerian donors and their respective ABO and Rh blood groups. There was also no correlation between the growth effect of individual serum samples and their respective membrane immunofluorescence reaction on OB3 cells. Thus, a serum sample with a low growth index did not necessarily give a positive membrane immunofluorescence reaction.

### DISCUSSION

The knowledge gained in the growth experiments is closely similar to that reported in an earlier study in which another Burkitt tumour cell line (Strain

OB1) and a different series of sera from adult Nigerians and Burkitt tumour patients were tested, (Osunkoya, 1966).

It is likely, as has already been suggested, that several factors particularly of a nutritional nature are involved, in addition to probable immunological processes, in the manifestation of the effect of a serum sample on the growth of Burkitt tumour cells in vitro. The active participation of immune or related processes is however suggested by the trend observed by Ngu *et al.* (1966) between the 1gM level of some serum samples and their growth effects on a Burkitt tumour cell line (OB6).

It is well known in tissue culture work that some human and animal sera contain thermolabile substances of as yet unknown nature, which make such sera " toxic " to cell growth *in vitro*. An immunological phenomenon is still to be excluded in the search for a satisfactory explanation to account for the action of these " toxic " sera. Sera heated at 56° C. for 30 minutes regularly have a lower growth index than the respective uninactivated serum when tested on Burkitt tumour cell lines, (unpublished).

The result of the immunofluorescence tests on OB3 cells cultured in sera from various individuals are of interest, and essentially confirms the results of Klein *et al.* (1966) concerning East Africans. Sera from Swedish blood donors tested by these workers were however negative.

Several conclusions are readily drawn from the set of figures shown in Table II. We can now say that (i) cells that have been propagated in tissue culture for more than nine months after isolation from a patient suffering from Burkitt tumour bear on their surface moieties perhaps of an antigenic character, which react with human serum globulins to such a degree as to be detectable by the indirect (sandwich) fluorescent antibody reaction. (ii) Some Nigerians as well as North Americans do possess serum globulins which have affinity for the surface of these cells. (iii) Blood donors in New York have a lower incidence of individuals possessing such globulins as compared with Nigerian blood donors ($\chi^2 = 9{\cdot}134$. $p < 0{\cdot}005$). (iv) Burkitt tumour patients show more tendency of having this type of globulin than sick Nigerian children of comparable age ($\chi^2 = 6{\cdot}306$. $p < 0{\cdot}025$). (v) Apparently a higher proportion of Americans 2 years resident in Nigeria produce this serum globulin, than a comparable newly arrived group ($p = 0{\cdot}064$). (vi) The New York American Caucasian is as likely to possess this globulin as the New York Negro.

The interpretation of these findings can, at this stage of our studies, be only a matter of conjecture. If the positive " ring " test (Moller, 1961) is a genuine index of an immunological reaction, then it would appear that antibodies to surface antigens on OB3 cells are present in detectable concentration in a little less than half of Nigerian blood donors, and about a fifth of New York blood donors. The incidence of positive individuals is quite low in Nigerian children unless they are suffering or have suffered from Burkitt tumour.

The nature of the reactant antigens on the surface of Burkitt tumour cells is not known. The probability that the antigens are Burkitt tumour specific has much to commend itself, but several other possibilities have to be considered for exclusion, particularly if cell lines are used in the search for experimental proof of this surmise.

The non-correlation of positive sera with ABO and rhesus blood group of donors has been mentioned already. The combined incidence of Anti-M, N and S

haemagglutinins in the sera of Nigerian blood donors is less than 10% (Luzzatto personal communication), a figure too low to account for an incidence of over 40% positive sera in the Nigerian blood donors studied. Klein *et al.* (1966) were also able to exclude the involvement of blood group antigens in the analysis of their results.

The increasing reports of contamination of cell cultures by pleuropneumonia-like organisms (PPLO), with their characteristic tendency to aggregate on cell surfaces, brings this class of organisms under consideration in any immunological tests involving the cell surface of cultured cells (Edwards and Fogh, 1960; Kraemer *et al.*, 1963; Fogh and Fogh, 1964; Holmgren find Payne, 1966). Electron microscopic examination have so far failed to demonstrate Mycoplasma (PPLO) in OB3 cultures (Dalton, personal communication).

The attractive hypothesis of virus-induced, insect-vectored aetiology of Burkitt tumour (Davies, quoted by Burkitt, 1962a; Burkitt, 1962b; Stanley, 1966) has given much impetus to the search for viral agents in Burkitt tumour materials. Herpes-type virus particles have been demonstrated in several Burkitt tumour cell lines (Epstein *et al.*, 1964, 1965; Stewart *et al.*, 1965; Rabson *et al.*, 1966). The point in time at which the cells were infected by this viral agent, as well as its importance if any, in the aetiogenesis of Burkitt tumour are still subjects of controversy.* [No virus particles were seen in OB3 cells by electron microscopy, at the time they were used in the present study (Dalton, personal communication).] The role of several other groups of viruses which have been isolated from Burkitt tumour materials is not clear. Herpes simplex virus has been isolated directly from tumour biopsy material in a few out of several attempts (Woodall *et al.*, 1965; Simons and Ross, 1965. Dalldorf and Bergmaini (1964) isolated filterable antigenically-related agents now known to be mycoplasma from tumour, bone marrow and faeces of six out of eight East African children suffering from Burkitt tumour. Lastly, reovirus type 3, a virus whose exact role, if any, in the pathogenesis of murine lymphoma is as yet not clear, (Stanley, 1966; Stanley *et al.*, 1966(b)) have also been isolated from Burkitt tumour biopsy materials, including ascitic fluid (Bell *et al.*, 1966; Bell, 1966).

There is striking similarity between the results of the present investigations and reports on some serological investigations carried out in East Africa in which it was shown that 53 out of 72 (73%) Burkitt tumour patients had demonstrable antibodies to reovirus type 3 as compared with 12 out of 65 (18%) healthy East African children, a highly significant difference, (Bell, 1966).

While awaiting the characterisation of the cell surface antigens on OB3 and other Burkitt tumour cells, the results of the present study would suggest that some of these antigens may be related to the pathogenesis of Burkitt tumour, since it is children with this tumour as well as their relatives that produced the highest incidence of positive sera. This conclusion supports the concept of Burkitt tumour being a rare aberrant response to an infective, probably viral agent which occurs commonly in tropical Africa. (Haddow and McCallum, 1962; Burkitt, 1963). It is possible that this agent or a closely related variant also occurs in and around New York. It is considered that ease of perpetuation and transmission, and more frequent exposure to the agent may account for the relatively higher incidence of Burkitt's tumour in Africa.

* Two months after this paper was submitted for publication, occasional herpes-like virus particles were seen in a few OB3 cells by electron microscopy. (V. H. Zeve, 1966, personal communication).

SUMMARY

(1) Sera from various individuals were tested on cells from a Burkitt tumour cell line (OB3), for effect on growth of these cells *in vitro*, and for presence of antibodies to surface antigens on these cells.

(2) Sera from Burkitt tumour patients, particularly those in long-term remission, have relatively low growth promoting effects on OB3 cells.

(3) Using the indirect immunofluorescence reaction, antibodies to surface antigens on living OB3 cells were detected in serum samples from a little less than half of Nigerian blood donors, as compared with a fifth of New York blood donors tested ($p < 0{\cdot}005$). There was no significant difference in the incidence of positive sera between Negro and Caucasian New York blood donors. The incidence of positive sera was quite low in sick Nigerian children unless they are suffering or have suffered from Burkitt tumour ($p < 0{\cdot}025$). Although a higher proportion of young Americans resident in Nigeria produced positive sera than a comparable newly arrived group, and although it would appear that the incidence of positive sera was highest in the parents of Burkitt tumour patients the small numbers tested in these groups preclude statistical significance.

(4) The nature of the surface antigens on Burkitt tumour cells is discussed in the light of the above findings. It is considered that these antigens are related to the aetiogenesis of Burkitt tumour, and may be infective in origin.

(5) There was no correlation between the growth effect of serum samples and their respective membrane immunofluorescence results.

I am grateful to Prof. G. M. Edington and Prof. V. A. Ngu for their advice and encouragement; Prof. A. Lucas, Drs. L. Luzzatto and O. A. Okubadejo for helpful criticism; Drs. Burchenal, Oettgen and Friedland and several of mycolleagues for supplying some of the serum samples tested; and the staff of the Medical Illustration Unit, University College Hospital, Ibadan for preparation of figures. The technical assistance of Miss F. Mottram is gratefully acknowledged.

This work was supported partly by a fellowship from the Rockefeller Foundation, and a grant from the British Empire Cancer Campaign for Research.

### REFERENCES

Bell, T. M.—(1966) in U.I.C.C. Symposium on Chemotherapy of Burkitt tumour Kampala. In press.

Bell, T. M., Massie, A., Ross, M. G. R., Simpson, N. I. H. and Griffin. E.—(1966) *Br. med. J.*, i, 1514.

Burkitt, D.—(1962*b*) *Nature, Lond.*, **194**, 132.

Burkitt, D.—(1963) *Int. Rev. Path.*, **2**, 132.

Burkitt, D.—(1966) in U.I.C.C. Symposium on Chemotherapy of Burkitt tumour. Kampala. In press.

Burkitt, D., Hutt, M. R. S. and Wright, D. H.—(1965) *Cancer, N.Y.*, **18**, 399.

Dalldorf, G. and Bergamini, F.—(1964) *Proc. natn. Acad. Sci. U.S.A.*, **51**, 263.

Davies, J. N. P., quoted by Burkitt, D.—(1962*a*) *Postgrad. med. J.*, **38**, 71.

Edwards, G. A., and Fogh, J.—(1960) *J. Bact.*, **79**, 267.

Epstein, M. A., Achong, B. G., and Barr, Y. M.—(1964) *Lancet*, i, 702.

Epstein, M. A., Henle, G., Achong, B. G., and Barr, Y. M.—(1965). *J. exp. Med.*, **121**, 761.

Fogh, J. and Fogh, H.—(1964) *Proc. Soc. exp. Biol. Med.*, **117**, 899.

Gorer, P. A. and O'Gorman, P.—(1956) *Transplantn Bull.*, **3**, 142.
Haddow, A. J. and McCallum, D.—(1962) Annual report of the East African Virus Research Institute.
Holmgren, N. B. and Payne, F. C.—(1966) *J. natn. Cancer Inst.*, **36**, 355.
Klein, G., Clifford, P., Klein, E. and Stjernsward, J.—(1966) in U.I.C.C. Symposium on Chemotherapy of Burkitt Tumour. Kampala. In press.
Kraemer, P. M., Defendi. V. Hayflick, L. and Manson, L. A.—(1963) *Proc. Soc. exp. Biol. Med.*, **112**, 381.
Moller, G.—(1961) *J. exp. Med.*, **114**, 415.
Ngu, V. A., (1966) in U.I.C.C. Symposium on Chemotherapy of Burkitt tumour. Kampala. In press.
Ngu, V. A., Macfarlane, H., Osunkoya, B. O. and Udeozo, I. O. K.—(1966) *Lancet*, ii, 414.
Osunkoya, B. O.—(1965) *Br. J. Cancer*, **19**, 749.
Osunkoya, B. O.—(1966) in U.I.C.C. Symposium on Chemotherapy of Burkitt tumour. Kampala. In press.
Rabson, A. S., O'Conor, G. T., Baron, S., Whang, J. J. and Legallias, F. Y.—(1966) *Int. J. Cancer*, **1**, 89.
Simmons, P. J., and Ross, M. G. R.a(1965) *Eur. J. Cancer*, **1**, 135.
Stanley, N. F.—(1966) *Lancet*, i, 961.
Stanley, N. F., Walter, M. N. I., Leak, P. J. and Keast, D.—(1966*a*) *Proc. Soc. exp. Biol. Med.*, **121**, 90.
Stanley, N. F., Papadimitriou, J. M., and Epstein, M. A.—(1966*b*) *Br. med. J.*, ii, 767.
Stewart, S. E., Lovelace, E., Whang, J. J. and Ngu, V. A.—(1965) *J. natn. Cancer Inst.*, **34**, 319.
Woodall, J. P., Williams, M. C., Simpson, D. I. H., and Haddow, A. J.—(1965) *Eur. J. Cancer*, **1**, 137.